# Chronic disease prevention in primary care: how and when will genomics impact?

**DOI:** 10.3399/bjgp14X680401

**Published:** 2014-06-30

**Authors:** Fiona M Walter, Jon Emery, Hilary Burton

**Affiliations:** The Primary Care Unit, Department of Public Health & Primary Care, University of Cambridge, Cambridge, UK, & General Practice and Primary Care Academic Centre, University of Melbourne, Australia.; General Practice and Primary Care Academic Centre, University of Melbourne, Australia, and The Primary Care Unit, Department of Public Health & Primary Care, University of Cambridge, Cambridge, UK.; Cambridge, UK.

Over the past few years there has been considerable investment in genomics (DNA-based) research, including the Prime Minister’s announcement of £100 million to sequence 100 000 whole genomes.^1^ Despite so much hype about the anticipated impact of genomics on our understanding of susceptibility to cancer and other chronic diseases, new interventions have only recently begun to emerge.^2^ When and how are these advances likely to impact on primary care’s key role in preventing and treating common chronic diseases?

## CHRONIC DISEASE PREVENTION IN PRIMARY CARE

Chronic disease prevention in primary care aims to identify individuals at increased risk who can then be given tailored disease prevention advice including lifestyle modification, disease screening, or preventive treatment. Current approaches to chronic disease risk assessment use a combination of factors including demographics, family history, lifestyle, physiological measurements, and biomarkers. GPs in the UK are familiar with using electronic risk calculators to provide a personal risk score, such as the QRISK2 algorithm for primary prevention of cardiovascular disease.^3^ The family medical history currently makes an important contribution to risk assessment for many diseases as the risk of a number of cancers, type 2 diabetes, and ischaemic heart disease are all increased in the presence of a family history; this risk increases with more affected relatives.^4^ Family history can be viewed as a proxy for genetic predisposition as well as environmental and lifestyle factors, and remains the most clinically relevant genetic risk tool for GPs.

## RECENT GENOMICS ADVANCES

While epidemiological studies have elucidated the most important risk factors for disease over the past half century, this work largely considered the population as homogeneous, with risk factors acting uniformly across the population in relation to diseases identified. Recent genomic advances have provided new insights such as that the diseases themselves are heterogeneous.^5^ There is also increasing evidence that an individual’s basic genetic background interacts with environmental exposures and modulates responses to preventive interventions. Such knowledge has started to address important areas such as why some smokers do not get various forms of cancer,^6^ and why some people may be particularly prone to alcohol addiction.^7^

## GENOMICS AND CHRONIC DISEASE RISK

In the light of these advances, DNA-based risk assessment is increasingly being proposed as a means of determining chronic disease risk. Recent work undertaken by the European Commission funded multicentre Collaborative Oncological Gene-environment Study (COGS) on genetic variation and breast cancer risk has provided proof of principle that testing for genetic variants could be used to stratify the population so that those at higher risk might receive earlier or more frequent breast screening, while those at lower risk might even be recommended to forgo screening.^8^ Stratification of the programme in this way could mean that the potential harms of mammography (such as unnecessary biopsies) could be minimised while still detecting most women with breast cancer. Similar work is under way on cardiovascular disease, whereby genetic testing might augment current risk scores, enabling people at risk to be identified earlier before they develop phenotypic markers of risk, or with more precision, thus avoiding administration of statins to those who are unlikely to benefit. Proponents suggest that individuals who are low risk would then not be inconvenienced, treated, or even potentially harmed by preventive interventions such as statins that provide minimal benefit, and that resources could be used more efficiently.

## PERSONALISED DISEASE PREVENTION IN PRIMARY CARE

Given adequate resourcing, a range of DNA-based tests could be used to fine-tune the current approach to prevention, and provide more tailored chronic disease prevention in primary care. This may be further enabled with near-patient testing for a particular set of genetic variants, undertaken in the surgery. Applied in this way, genomics could then support tailored cancer screening, the integration of genomics data with family history, sociodemographic, behavioural, and environmental risk factors into risk assessment tools, and even pharmacogenetically-informed prescribing such as for nicotine replacement treatment. However, there are still a number of issues that remain important to address before the incorporation of any of these approaches into routine general practice.

## PRIMARY CARE PREPARATION FOR GENOMICS

What preparation might GPs need? The answer, in our mind, is not very much, as most of the genomics-based approaches will be delivered using GPs’ current expertise in communicating with patients about disease prevention, lifestyle and behaviour change. A detailed understanding of genetic variants and their additional contribution to risk profiling may not be required, assuming the validity of the risk assessment. We will need to incorporate discussions about the impact of DNA testing, including that a person’s level of risk could be determined at an earlier age and before phenotypic markers of risk or disease are detectable by biomarkers. The importance of adopting appropriate lifestyle changes will still need to be stressed. Where prevention options include interventions such as screening or drug treatment we will need to help patients understand that such interventions are not universally beneficial; those whose genetic background puts them at lower risk may need to understand the potential for harm in these activities. DNA-based risk assessment also raises ethical issues for primary care, particularly with near-patient testing, such as whether the genetic samples and test results will be stored for future reference or discarded. If stored, consent and access issues will become important, particularly around employment, insurance, and research by academic or commercial organisations.

## IMPACT OF GENOMICS ON CHRONIC DISEASE PREVENTION IN PRIMARY CARE

As genomics-based approaches begin to be implemented both within health services and, increasingly, through direct-to-consumer testing, there is still much research to be undertaken. In a recent issue Middlemass and colleagues reported a qualitative study investigating the introduction of genetic testing for cardiovascular disease in primary care.^9^ While they found that the testing was acceptable, they also highlighted that:

‘... *in some participants the genetic test results would reinforce healthy behaviour while others were falsely reassured.*’

They emphasised that it will be important to build on the patient’s motivation to undertake genetic testing both for themselves and their families; to understand how patients deal with conflicting findings between genetic and conventional cardiovascular risk assessment; to learn how to promote responses that reinforce healthy behaviour change and reduce false reassurance; and to look at the potential benefit of genetic testing for younger people who may have more to gain from risk reducing behaviour.

Educational strategies will also be of value to primary care clinicians. The NHS National Genetics and Genomics Education Centre has developed an e-learning programme for the RCGP entitled ‘Genetics in Primary Care’.^10^ The website also features other relevant learning and teaching opportunities around genomics in health care. This will be an integral part of the overall strategic programme for genomics-related education and training interventions for the NHS workforce, which is being led by the newly created Genomics Advisory Board under Health Education England. We believe that it will be important to grasp current opportunities to expand professional education in genetics beyond the current remit, which is largely focused on rare heritable disease. Primary care will soon need to integrate genomics into its consideration of chronic disease prevention and start to prepare for what is likely to be less than a decade away.
